# Identification of the Role and Clinical Prognostic Value of Target Genes of m6A RNA Methylation Regulators in Glioma

**DOI:** 10.3389/fcell.2021.709022

**Published:** 2021-09-13

**Authors:** Peilin Cong, Tingmei Wu, Xinwei Huang, Huazheng Liang, Xiaofei Gao, Li Tian, Wanrong Li, Aiwen Chen, Hanxi Wan, Mengfan He, Danqing Dai, Zhen Li, Lize Xiong

**Affiliations:** Translational Research Institute of Brain and Brain-Like Intelligence, Shanghai Fourth People’s Hospital, School of Medicine, Tongji University, Shanghai, China

**Keywords:** m6A RNA methylation regulators, methylation modification, glioma, glioblastoma, bioinformatics, cancer, METTL3, prognostic marker 2

## Abstract

m6A RNA methylation regulators can regulate the growth, progression, and invasion of glioma cells by regulating their target genes, which provides a reliable support for the m6A regulator–target axes as the novel therapeutic targets and clinical prognostic signature in glioma. This study aimed to explore the role and prognostic value of m6A RNA methylation regulators and their targets. Expression profiles and clinicopathological data were obtained from the Chinese Glioma Genome Atlas (CGGA), The Cancer Genome Atlas (TCGA), Gene Expression Omnibus (GEO), and Clinical Proteome Tumor Analysis Consortium (CPTAC) datasets. Differential expression and correlation analyses were performed between normal and glioma tissues at mRNA and protein levels. Univariate Cox regression, survival, and Lasso Cox regression analyses were conducted to identify and establish the prognostic gene signature. Kaplan–Meier curve, multivariate Cox regression analysis, and ROC were utilized to evaluate the prognostic capacity of the prognostic gene signature. The correlation analysis, systematic bioinformatics analysis, and cell experiment were performed to further understand the potential underlying molecular mechanisms and drug sensitivity. Our results suggested that IGF2BP2, KIAA1429, METTL16, and METTL3, as well as 208 targets are involved in the occurrence of glioma, GBM, and LGG. YTHDF1 and 78 targets involved the occurrence of glioma and GBM, not LGG, among which 181 genes were associated with overall survival. From other findings and our cell experiment results, we demonstrated that METTL3 can activate Notch pathway and facilitate glioma occurrence through regulating its direct targets NOTCH3, DLL3, and HES1, and Notch pathway genes may serve as the potential treatment targets for glioma. Our study established and validated a seven-gene signature comprising METTL3, COL18A1, NASP, PHLPP2, TIMP1, U2AF2, and VEGFA, with a good capability for predicting glioma survival, which may guide therapeutic customization and clinical decision-making. These genes were identified to influence 81 anticancer drug responses, which further contributes to the early phase clinical trials of drug development.

## Introduction

**FIGURE 1:**
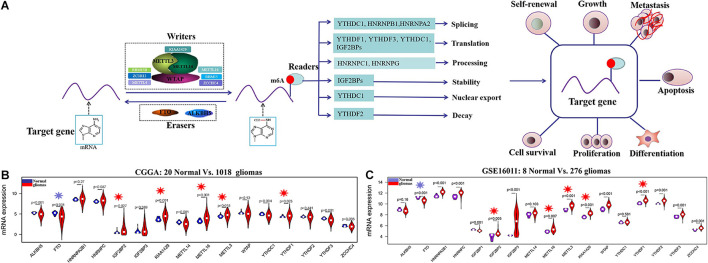
The regulation mechanism of m6A RNA methylation regulators in human cancers and their expression profiles in glioma at mRNA level. **(A)** The target gene is mediated by writers containing METTL3, METTL14, METTL16, WTAP, KIAA1429, RBM15B, ZC3H13, etc.; erasers ALKBH5 and FTO; and reader proteins YTHDFs, YTHDCs, HNRNPB1, HNRNPC, HNRNPA2, HNRNPG, IGF2BPs, and so on. Aberrant regulation of m6A RNA methylation regulators in their target mRNAs may activate or inhibit tumor occurrence and progression by dsyregulating tumor cell proliferation and differentiation tumorigenesis, invasion, and metastasis. **(B,C)** The mRNA expression status of m6A RNA methylation regulators in the merged CGGA dataset (Normal = 20; Glioma = 1018) and GSE16011 dataset (Normal = 8; Glioma = 276).

N6-methyladenosine (m6A) RNA methylation, first discovered in the 1970s, is the most prevalent dynamic and reversible epigenetic modification in mRNAs (Desrosiers et al., 1974). m6A RNA methylation on target gene is installed by methyltransferases (writers) containing METTL3, METTL14, WTAP, METTL16, ZCCHC4, and KIAA1429, among others, and removed by demethylases (erasers) composed of FTO and ALKBH5. The function of m6A-modified targets is executed by m6A binding proteins (readers) through binding to m6A directly or indirectly, including YTH N6-methyladenosine RNA-binding proteins, YTH domain-containing proteins, IGF2BPs, and HNRNP protein families (Tong et al., 2018). These writers, erasers, and readers, serving as m6A RNA methylation regulators, can influence mRNA of target gene at different levels, including nuclear export, translation, splicing, stability, and decay (Lan et al., 2019; Huang et al., 2020; Nombela et al., 2021) and have important influences in diverse physiological and pathological processes, such as cell proliferation and differentiation, oncogenic protein expression, and the proliferation, survival, initiation, and progression of tumor cell (Figure 1A; Chen et al., 2019; Lan et al., 2019; Du et al., 2020; Huang et al., 2020; Nombela et al., 2021).

Glioma is one of the most common brain cancers (Dixit et al., 2021), including low-grade glioma (LGG) (WHO grade II) and glioblastoma (GBM) (WHO grade IV) (Parsons et al., 2008; Wen and Kesari, 2008). Although surgery, radiotherapy, and alkylating chemotherapy have been widely utilized (Wirsching et al., 2016), the clinical prognosis of glioma patients is not good after diagnosis (Van Meir et al., 2010; Du et al., 2020). Therefore, exploring the novel effective therapeutic targets and prognostic biomarkers for glioma is imperative. Recently, strong evidence suggested that m6A RNA methylation regulators including METTL3, METTL14, FTO, and YTHDF2 can regulate the self-renewal, tumorigenesis, growth and progression, and invasion of glioma cell by altering mRNA expression levels of their target genes (e.g., ADAM19, MYC, VEGFA, NCOR2, and HIVEP2) (Cui et al., 2017; Li F. et al., 2019; Dixit et al., 2021; Fang et al., 2021; Huff et al., 2021), which provides a reliable support for the m6A RNA methylation regulator–target gene axes as specific and novel therapeutic targets and clinical prognostic biomarkers in glioma.

In this study, we carried out a systematic analysis for m6A RNA methylation regulators and their targets in the mRNA and protein datasets to identify their roles in the treatment and clinical prognosis for glioma patients.

## Materials and Methods

### Data Acquisition and Preprocessing

The mRNA-seq and mRNA-array datasets and corresponding clinical information of glioma were obtained from The Cancer Genome Atlas (TCGA) database of the UCSC Xena public datasets^1^, Chinese Glioma Genome Atlas (CGGA) database^2^, and Gene Expression Omnibus (GEO) database^3^. Proteomic data of GBM patients were downloaded from Clinical Proteome Tumor Analysis Consortium (CPTAC) database^4^. mRNA datasets of CGGA included mRNA-seq_20 (20 normal brain tissues), mRNA-seq_325 (325 glioma tissues), mRNA-seq_693 (693 glioma tissues), and mRNA-array_301 (301 glioma tissues). We merged the 20 normal and 1,018 glioma RNA-seq data of CGGA and then normalized and removed their batch effect utilizing normalizeBetweenArrays and ComBat functions (Leek et al., 2012). In these RNA-seq data of CGGA, 1,018 glioma patients include 291 LGG, 388 GBM, and 339 other glioma types. The mRNA dataset of TCGA consisted of 5 normal, 168 GBM, and 538 LGG tissues. mRNA-array data of GSE108474 and GSE16011 were downloaded from the GEO database. GSE108474 contained 435 glioma patients composed of 208 GBM, 83 LGG, and 144 other glioma types and the corresponding clinical information. GSE16011 included 8 normal brain tissues and 276 glioma tissues including 159 GBM and 24 LGG. Protein data of CPTAC datasets contained 10 normal and 100 GBM tissues. Moreover, Gene Expression Profiling Interactive Analysis 2 (GEPIA 2) database had 207 GTEx normal tissues and 168 GBM and 518 LGG tissues.

### The Collection of Validated and Potential Targets of m6A RNA Methylation Regulators

The validated and potential targets of m6A RNA methylation regulators were obtained from the m6A2Target database^5^, which integrates various validated and potential m6A targets proved *via* high-throughput sequencing, including RIP-seq, ChIP-seq, RNA-Seq, and m6A-Seq (Deng et al., 2020).

### Identification of Differentially Expressed m6A RNA Methylation Regulators and Targets in Glioma

We performed a differential gene expression analysis for RNA-seq data in CGGA, TCGA, GSE16011, and GEPIA 2 database, respectively, and conducted a differential protein expression analysis in CPTAC. The R package limma (version 3.44.1) (Ritchie et al., 2015) and wilcox test were used to identify differentially expressed genes and proteins between normal and glioma samples. The false discovery rate (FDR) value <0.05 was applied as the cutoff for screening. In order to further identify the expression status of differentially expressed m6A RNA methylation regulators and targets in other cancer types, we performed a differential expression analysis for them by the GSC Gene Set Cancer Analysis (GSCA) database^6^, a multi-omics analysis server containing various functional modules, such as differential expression and the survival analyses (Liu et al., 2018). The |fold change| >1.5 and FDR value <0.05 were applied as the cutoff for screening.

### Correlation Analysis Between m6A RNA Methylation Regulators and Targets

R packages tidyr, dplyr, ggExtra, and ggstatsplot were applied to determine the cross-talk between m6A RNA methylation regulators and targets in the CGGA, TCGA, GSE16011, and CPTAC datasets, respectively. The |Cor| >0.20 and *p*-value < 0.01 were considered as statistically significant. The interactions were visualized *via* Cytoscape 3.7.1 (Otasek et al., 2019).

### Bioinformatic Analysis

The protein--protein interaction (PPI) network analysis was utilized to identify the interactions between m6A RNA methylation regulators and targets using the STRING database^7^. Kyoto Encyclopedia of Genes and Genomes (KEGG) and Gene Ontology (GO) enrichment analyses for differentially expressed genes were conducted using R packages clusterProfiler, org.Hs.eg.db, enrichplot, and ggplot2. The *q*-value < 0.05 was considered as the cutoff. To better understand the roles of the critical m6A RNA methylation regulators and targets in cancers, we performed a correlation analysis of gene-cancer pathway by GSCA (Liu et al., 2018). microRNA--mRNA interaction analysis was performed *via* starBase v3.0 database^8^ (Li et al., 2014).

### Survival Analysis of m6A RNA Methylation Regulators and Targets

Four glioma mRNA datasets of CGGA, namely, mRNA-seq_325, mRNA-seq_693, the merged mRNA-seq_1018, and mRNA-array_301, were used to identify m6A RNA methylation regulators and targets associated with the occurrence and overall survival of glioma. Univariate Cox proportional hazards regression and survival analyses for differentially expressed genes were performed utilizing R packages survival and survminer. We conducted a survival analysis for these genes *via* GSCA to further determine the association of glioma-related m6A RNA methylation regulators and targets with overall survival of other cancer types. *p* < 0.05 was applied the cutoff.

### Construction and Validation of Prognostic Gene Signature

Lasso-penalized Cox regression analysis was conducted to further identify the prognostic gene signature in seven sets of mRNA datasets of glioma, GBM, or LGG (Tibshirani, 1997). Based on the R package glmnet, a prognostic gene signature of glioma patients was obtained on the basis of a linear combination of the regression coefficient that is derived from the Lasso Cox regression model coefficients (β) multiplied with its expression level. The gene signature risk score for each patient was calculated by the formula: βGene1 ^∗^ expression level of Gene1 + βGene2 ^∗^ expression level of Gene2 + βGene3 ^∗^ expression level of Gene3 + ⋯ + βGeneN ^∗^ expression level of GeneN. Glioma patients were divided into high- and low-risk subgroups according to the optimal cutoff of the prognostic gene signature.

Kaplan–Meier plot was performed to compare the overall survival between two risk subgroups utilizing the R package survival. The time-dependent receiver operating characteristic (ROC) curve was used to assess the predictive value of the prognostic gene signature for overall survival using R package survivalROC. The two-sided *p* < 0.05 was applied as the cutoff. Multivariate Cox proportional hazard regression analyses for four CGGA datasets with complete information were conducted to determine what clinical factors and whether gene signature could be used as prognostic indicators for the overall survival of glioma. *p* < 0.05 was applied as statistically significant.

### Correlation Analysis Between Drug Susceptibility and the Expressions of Glioma-Related Genes

RNA-seq and compound activity (DTP NCI-60) data were downloaded from the CellMiner database, which is a genomic and pharmacologic database to explore transcript and drug activity in the NCI-60 cell line set containing data from the Cancer Cell Line Encyclopedia (CCLE), Cancer Therapeutics Response Portal (CTRP), and Genomics of Drug Sensitivity in Cancer (GDSC) cell line datasets (Reinhold et al., 2012). R packages impute, limma, ggplot2, and ggpubr were utilized to identify the associations between drug susceptibility and genes. All included drugs were approved by the FDA or validated by clinical trial. |Cor| > 0.30 and *p* < 0.01 were applied as the cutoff.

### Cell Culture and Cell Transfection With siRNA

The human GBM cell lines U87MG and U118MG were purchased from the American Type Culture Collection (ATCC). U87MG and U118MG was maintained in Minimum Essential Medium (MEM) and Dulbecco’s modified Eagle’s medium (DMEM), respectively, supplemented with 10% fetal bovine serum (Gibco, United States), 100 μg/ml streptomycin, and 100 U/ml penicillin, at 37°C in 5% CO_2_. Three small interfering RNAs (siRNAs) against METTL3 were used to inhibit METTL3 expression. siCrtl was used as a control. METTL3 siRNA sequences were shown in Supplementary Table S1. All siRNAs were designed and chemically synthesized by Obio Technology (Obio Technology Co. Ltd., China). siRNAs were transfected into U87MG and U118MG using X-treme GENE^TM^ HP DNA Transfection Reagent (Roche, Switzerland) according to the manufacturer’s instructions. Cells were harvested for RNA and protein extraction assay at 48 h after the transfection.

### RNA Extraction, Reverse Transcription, and qRT-PCR

Total RNA was extracted using Trizol reagent (Thermo Fisher Scientific, United States), and reverse transcription was performed with Primer Script RT reagent kit (Vazyme, China). Then, qRT-PCR was performed using a SYBR Green master mix (Vazyme, China) on the Quant Studio 1 real-time PCR system (Thermo Fisher Scientific, United States). Primer sequences used are shown in Supplementary Table S1. GAPDH was used as a housekeeping control. The 2^–ΔΔCt^ method was utilized to assess the relative expression levels of genes.

### Western Blotting

Cells were lysed using M-PER Mammalian Protein Extraction Reagent lysis buffer (Thermo Fisher Scientific, United States) and protease inhibitor cocktail (Bimake, China). The BCA protein assay Kit (Epizyme, China) was used to quantitate total protein levels. Proteins were separated by sodium dodecyl sulfate−10% polyacrylamide gel (SDS-PAGE) electrophoresis and transferred onto polyvinyl difluoride (PVDF) membranes (Millipore, United States). The membranes were blocked for 2 h at room temperature in TBST with 5% non-fat milk and incubated overnight at 4°C with primary antibodies including anti-β-actin (AF7018, 1:3,000, Affinity), anti-DLL3 (ab103102, 1:1,000, Abcam), anti-METTL3 (ab195352, 1:1,000, Abcam), anti-NOTCH3 (5278T, 1:1,000, Cell Signaling Technology), and anti-HES1 (11988S, 1:1,000, Cell Signaling Technology). The membranes were washed three times with TBST and incubated with anti-rabbit IgG HRP-linked antibody (31466, 1:3,000, Thermo Fisher Scientific) in room temperature. Finally, the ChemiDoc XRS + system (Bio-Rad, United States) was used to detect protein signal by Immobilon Western Chemiluminescent HRP substrate (Millipore, United States). The quantifications were evaluated using ImageJ software.

### Cell Counting Kit-8 Assay

Cell proliferation was monitored using Cell Counting Kit-8 (CCK-8) assay (Biodragon, China) according to the manufacturer’s instructions. Briefly, cells in logarithmic growth stage were seeded into 96-well plates (1 × 10^4^ cells/well) and 10 μl of CCK-8 solution was added at each well. The absorbance at 450 nM was measured after incubation at 37°C for 2 h with a microtiter plate reader. Measurements were separately done at 0, 24, 48, and 72 h after transfection of siRNA.

### Statistical Analysis

All statistical analyses of cell experiments were performed with GraphPad Prism 8.0 software. Other statistical analyses were conducted with R.V.4.0.3. Spearman was calculated as the correlation between m6A RNA methylation regulators and targets. In the univariate and multivariate Cox regression analyses, the hazard ratio (HR) and 95% confidence interval (CI) were calculated to determine genes related to overall survival of glioma patients. The unpaired and two-tailed Student’s *t*-test was used to determine the statistical significance between two groups. The one-way ANOVA analysis with *post hoc* Tukey’s test was used when there are more than two groups. For CCK-8, the two-way ANOVA analysis was used to determine statistical significance among groups at different time points with Sidak’s multiple comparisons. All data were shown as the mean ± standard error of mean (SEM). Unless otherwise noted, *p* < 0.05 was applied as the cutoff.

## Results

### Identification of Differentially Expressed m6A RNA Methylation Regulators and Targets

**FIGURE 2:**
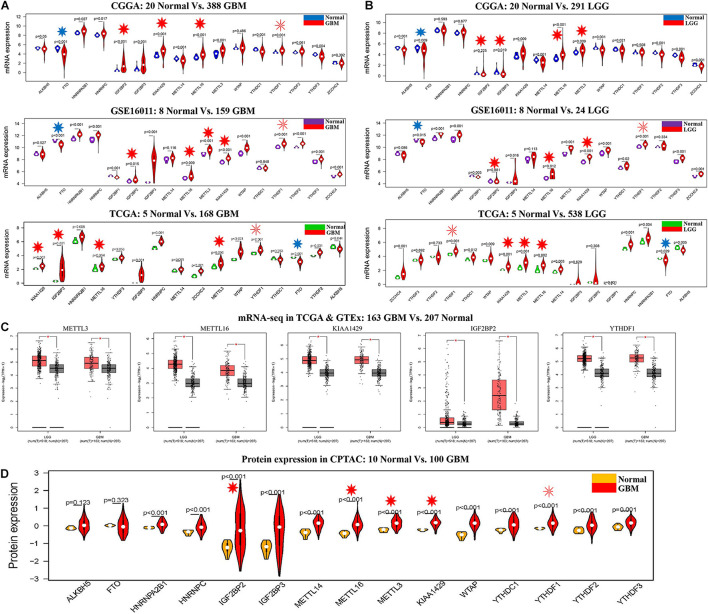
The expression profiles of m6A RNA methylation regulators in GBM and LGG. **(A)** mRNA expression status of m6A RNA methylation regulators in the CGGA, GSE16011, and TCGA datasets of GBM. **(B)** mRNA expression status of m6A RNA methylation regulators in the CGGA, GSE16011, and TCGA datasets of LGG. **(C)** mRNA expression status of m6A RNA methylation regulators in GBM and LGG by GEPIA 2 database. **(D)** Protein expression status of m6A RNA methylation regulators in the CPTAC dataset of GBM. *p < 0.05.

The clinical characteristics for TCGA, CGGA, GSE16011, GSE108474, and CPTAC datasets are summarized in Supplementary Table S2. A workflow and scheme of this study are shown in Supplementary Figure S1. To identify the key m6A RNA methylation regulators and targets related to glioma, GBM, and LGG, we performed a differentially expressed analysis for them at the mRNA or protein level, respectively. For glioma, we found that IGF2BP2, KIAA1429, METTL16, METTL3, and YTHDF1 are consistently upregulated at the mRNA level of CGGA and GSE16011 datasets (7.97E-05 < FDR < 0.04) (Figures 1B,C and Supplementary Table S3). Similarly, IGF2BP2, KIAA1429, METTL16, METTL3, and YTHDF1 also exhibited a consistent upregulation at both mRNA and protein levels in five GBM datasets (2.15E-19 < FDR < 4.43E-09) (Figures 2A,C,D and Supplementary Table S3). Except for YTHDF1, IGF2BP2, KIAA1429, METTL16, and METTL3 also had a consistent upregulation at mRNA level of four LGG datasets (2.15E-19 < FDR < 4.43E-09) (Figures 2B,C and Supplementary Table S3). In addition, FTO showed a consistent downregulation at the mRNA level of glioma, GBM, and LGG, but not at the protein level of GBM.

Based on the identified correlation of five m6A RNA methylation regulators with their targets in the m6A2Target database (Supplementary Table S4), we conducted a differentially expressed analysis for 39 validated and 19,322 potential targets in CGGA, TCGA, GSE16011, and CPTAC datasets, respectively. Twenty-one upregulated and three downregulated validated targets were determined at the mRNA level of two glioma datasets and three GBM datasets, and at the protein level of one GBM dataset. Among these targets, only ATP6V1A, DLL3, IKBKB, MEPCE, NOTCH1, NRF1, SFRP1, and SOX2 showed a consistent expression profile among gliomas, GBM, and LGG (Supplementary Table S3 and Supplementary Figure S2). A total of 262 potential targets were found to be significantly dysregulated at the mRNA level of two glioma datasets and three GBM datasets, and at the protein level of one GBM dataset, among which 200 targets had consistent expression profiles among glioma, GBM, and LGG (Supplementary Table S3). Taken together, these results indicated that IGF2BP2, KIAA1429, METTL16, and METTL3, as well as 208 targets are involved in the occurrence of glioma, GBM, and LGG. YTHDF1 and 78 targets involved the occurrence of glioma and GBM.

In order to further identify the expression status of these genes in other cancer types, we performed a differential expression analysis in pan-cancers by GSCA. As summarized in Supplementary Table S5, compared with normal samples, IGF2BP2 was significantly upregulated in nine cancer types and downregulated in two cancer types. METTL3 expression was significantly elevated in BLCA and LIHC. YTHDF1 showed an upregulation in COAD, STAD, and ESCA. Two hundred and eighty targets were upregulated or downregulated in 14 cancer types, among which BAX, CENPK, DLL3, and NOTCH3 showed a consistent upregulation in multiple cancers and glioma, indicating that these genes may play a carcinogenic role in a variety of cancers.

### Function Enrichment Analyses

To explore the function of the above glioma-related m6A RNA methylation regulators and targets, we performed KEGG and GO enrichment analyses. We found that these genes are implicated in 350 biological processes of the nervous system, including growth and development, differentiation, transport, and ensheathment of multiple brain cell types, 87 molecular functions, and 74 signal pathways (*q*-value < 0.05) (Supplementary Figure S3 and Supplementary Table S6). To further understand the effect of these genes on the occurrence of cancer, the association between these genes and 10 famous cancer-related pathways were analyzed on GSCA. As shown in Supplementary Figure 4A, these genes were correlated with activation or inhibition of multiple pathways. In GBM, we found that the high expressions of 25 genes activate or inhibit eight cancer-related pathways, namely, TSC/mTOR, hormone AR, hormone ER, DNA damage response, RTK, EMT, apoptosis, and cell cycle (FDR < 0.05) (Supplementary Figure S4B). Furthermore, the potential miRNA–mRNA regulatory network of these genes was evaluated by starBase v3.0. The results showed that expressions of METTL3, METTL16, KIAA1429, IGF2BP2, and YTHDF1 are mediated by multiple miRNAs (Supplementary Figure S4C).

### The Interactions of m6A RNA Methylation Regulators With Their Targets

**FIGURE 3:**
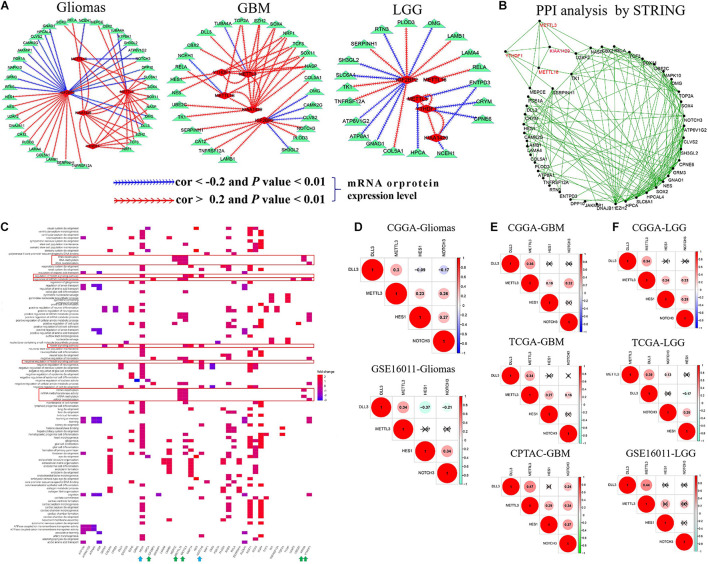
The cross-talk and biological functions of differentially expressed m6A RNA methylation regulators and their targets. **(A)** The identified correlation between five m6A RNA methylation regulators and targets in glioma, GBM, and LGG. Note: the |Cor| >0.20 and *p*-value < 0.01. **(B)** Validation of the cross-talk m6A RNA methylation regulators and targets by protein–protein interaction (PPI) network analysis. **(C)** Biological functions of differentially expressed m6A RNA methylation regulators and targets. **(D–F)** The interactions of METTL3 with DLL3, HES1, and NOTCH3 expressions in glioma, GBM, and LGG.

To confirm the interactions among five m6A RNA methylation regulators and corresponding targets, we performed correlation analyses for them in glioma, GBM, and LGG datasets. As summarized in Supplementary Table S7 and Figure 3A, METTL16 showed a consistent negative correlation with NCEH1 and NOTCH3 mRNA expressions (−0.39 < cor < −0.23, 1.67E-18 < *p* < 7.33E-05) and had a consistent positive association with the mRNA expressions of SOX4, DLL3, RELA, and KIAA1429 in two glioma datasets (0.23 < cor < 0.46, 1.63E-171 < *p* < 0.0001). The positive relationship between METTL16 and RELA expression was also identified in multiple GBM and LGG mRNA datasets and one GBM protein dataset (0.41 < cor < 0.58, 5.52E-71 < *p* < 0.004). In two glioma datasets, METTL3 exhibited a consistent negative relationship with TUBA4A mRNA expression (−0.35 < cor < −0.26, 9.53E-18 < *p* < 1.82E-09), while it had a positive association with the expressions of 15 genes, namely, DPP10, DLL3, CBX2, MEPCE, OMG, KIAA1429, SLC6A1, SOX4, SOX11, SOX2, YTHDF1, TCF3, EZH2, NASP, and NRF1 (0.21 < cor < 0.67, 1.16E-137 < *p* < 0.0003). The negative association between YTHDF1 and METTL3 was confirmed in multiple gliomas, GBM, and LGG datasets. In two glioma datasets, IGF2BP2 was negatively associated with the mRNA expression levels of 16 targets (−0.45 < cor < −0.20, 6.14E-51 < *p* < 0.008) and had a positive relevance with 12 targets (0.21 < cor < 0.59, 6.70E-102 < *p* < 0.004), among which the correlation of IGF2BP2 with SH3GL2, COL5A1, SERPINH1, and TNFRSF12A was also identified in multiple GBM and LGG datasets. Notably, m6A writers METTL3, METTL16, and KIAA1429 exhibited a cross-talk with the m6A reader YTHDF1 in gliomas, GBM, and LGG (Figure 3A). In addition, these results were further confirmed by PPI analysis that METTL3, METTL16, KIAA1429, and YTHDF1 interact with each other, and have direct or indirect interactions with their targets (Figure 3B).

We further explored their biological function using function enrichment analyses and found that METTL3, METTL16, KIAA1429, IGF2BP2, and YTHDF1 mainly regulate the methylation, destabilization, and metabolic process of RNA and mRNA, and influence the growth and development, differentiation, transport, and ensheathment of multiple brain cell types. Additionally, we observed that METTL3 is involved in the modulation of Notch signaling pathway (including METTL3 DLL3, HES1, and NOTCH3 genes) (*q*-value < 0.05) (Figure 3C), which plays an important role in tumorigenesis and cancer development (Li et al., 2017). Subsequently, we explored the potential regulation interactions between them utilizing correlation analysis in glioma, GBM, and LGG. As shown in Figures 3D–F, METTL3 was positively associated with DLL3, HES1, and NOTCH3 expressions in the CGGA glioma dataset, but not the GSE16011 glioma dataset (0.23 < cor < 0.34, *p* < 0.05). Similarly, this association was only determined at the mRNA level of the CGGA LGG dataset, but not the TCGA and GSE16011 LGG datasets (0.23 < cor < 0.34, *p* < 0.05). However, this association was identified in both mRNA and protein levels of GBM (0.19 < cor < 0.47, *p* < 0.05), which provides support for the previous observations that METTL3 knockdown can reduce the m6A methylation, transcript, and protein levels of DLL3, HES1, and NOTCH3 in the glioblastoma stem cell line and increase cell apoptosis (Supplementary Table S4).

### Knockdown of METTL3 Decreases DLL3, NOTCH3, HES1 mRNA, and Protein Levels Together With Cell Proliferation

**FIGURE 4:**
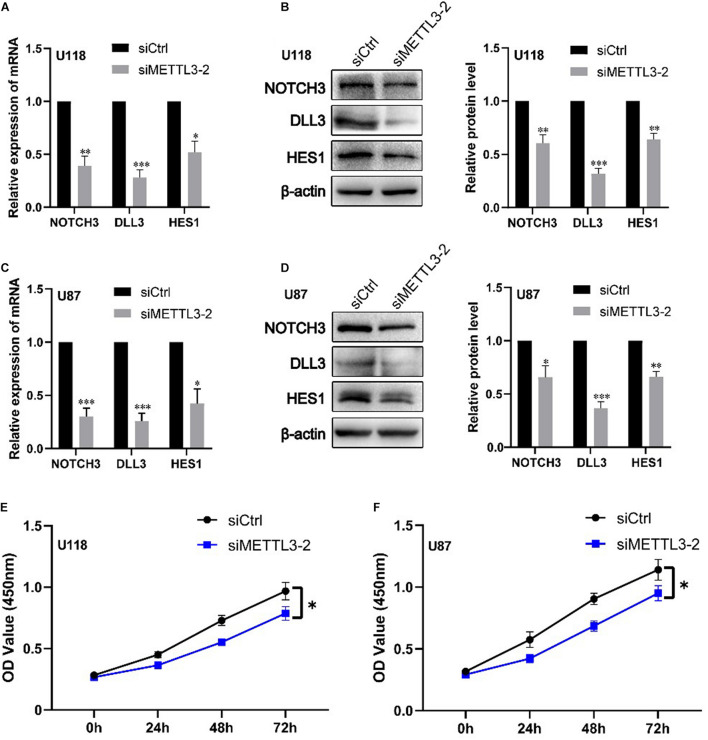
Knockdown of METTL3 decreases DLL3, NOTCH3, and HES1 mRNA and protein levels together with cell proliferation. **(A)** qRT-PCR analyses of the levels of NOTCH3, DLL3, and HES1 in METTL3 knockdown U118 cells (*n* = 3). **(B)** Western blot analyses of the levels of NOTCH3, DLL3, and HES1 in METTL3 knockdown U118 cells (*n* = 3). **(C)** qRT-PCR analyses of the levels of NOTCH3, DLL3, and HES1 in METTL3 knockdown U87 cells (*n* = 3). **(D)** Western blot analyses of the levels of NOTCH3, DLL3, and HES1 in METTL3 knockdown U87 cells (*n* = 3). **(E,F)** The proliferation ability of U87 and U118 cells was detected by CCK-8 detection. Data are presented as mean ± SEM. *represented comparison with siCtrl, ^∗^*p* < 0.05; ^∗∗^*p* < 0.01; ^∗∗∗^*p* < 0.001.

We further validated the regulation role of METTL3 in DLL3, NOTCH3, and HES1 and identify its influence in cell proliferation of glioblastoma cell lines *via* cell experiments. Firstly, we knocked down the expression of METTL3 using three different siRNAs against METTL3 in two glioblastoma cell lines (U87MG and U118MG). The knockdown efficiency of three independent siRNAs was detected by real-time qRT-PCR and Western blot. As shown in Supplementary Figure S5, siMETTL3-2 was selected for further experiments because it was the most effective siRNA. Subsequently, qRT-PCR and Western blot analyses showed that METTL3 knockdown significantly decreases the expression level of NOTCH3, DLL3, and HES1 compared with the control siRNA transfected in these two cell lines (*p* < 0.05) (Figures 4A–D). Furthermore, CCK8 assay showed that METTL3 knockdown inhibits glioblastoma cell proliferation at 72 h after transfection of siRNA (*p* < 0.05) (Figures 4E,F). These results indicated that the regulation role of METTL3 in DLL3, HES1, and NOTCH3 plays an important role in glioma, especially GBM.

### Identification of Survival-Related m6A RNA Methylation Regulators and Targets

To determine the prognostic value of five m6A RNA methylation regulators and their 286 targets in glioma and other cancer types, we performed survival and univariate Cox regression analyses in four sets of mRNA-seq or mRNA-array datasets, and conducted a survival analysis for these genes *via* GSCA. As summarized in Supplementary Table S8, 181 genes were found and validated to be consistently associated with overall survival in all mRNA expression datasets, among which the high expressions of 154 genes showed a good overall survival of glioma (HR < 1, *p* < 0.05), while the high expressions of 27 genes exhibited a poor overall survival of glioma (HR > 1, *p* < 0.05). By GSCA, we further evaluated that 179 of 181 glioma survival-related genes are related to overall survival of 27 other cancer types. Of the 181 genes, 97 genes were consistently associated with overall survival of LGG and glioma, while none was found to be associated with overall survival of GBM.

**FIGURE 5:**
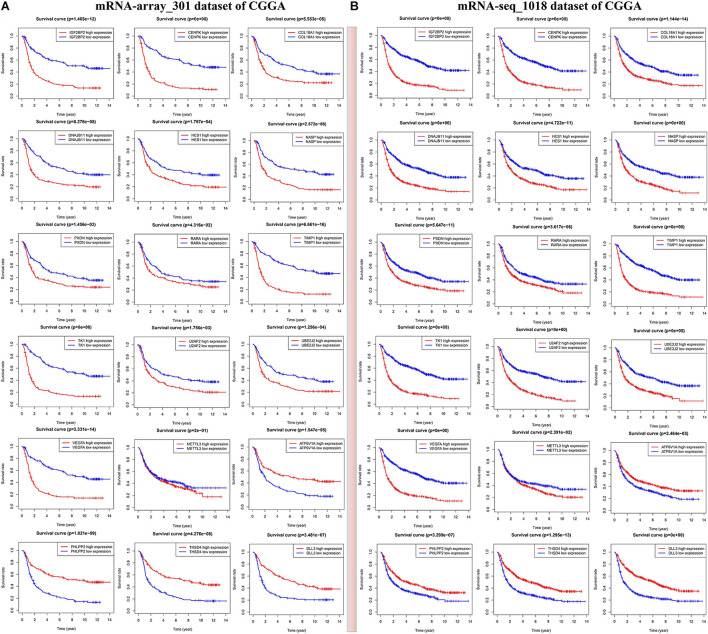
Identification of m6A RNA methylation regulators or validated targets associated with overall survival of glioma patients in two sets of CGGA mRNA datasets including mRNA-seq_1018 **(A)** and mRNA-array_301 **(B)**.

In the current study, we mainly focus on prognostic survival value of the five m6A RNA methylation regulators and 24 corresponding validated targets. In two CGGA mRNA datasets, we found that glioma patients with high expressions of METTL3, IGF2BP2, VEGFA, TIMP1, HES1, COL18A1, TK1, DNAJB11, CENPK, NASP, and U2AF2 have a shorter overall survival (HR > 1, *p* < 0.05) (Figure 5A and Supplementary Table S8), while those with high expressions of PHLPP2, ATP6V1A, and DLL3 show a good overall survival (HR < 1, *p* < 0.05) (Figure 5B and Supplementary Table S8). The above findings were also validated in two other CGGA mRNA datasets (Supplementary Figure S6), which indicates that the expressions of IGF2BP2 and METTL3 and the 12 corresponding targets might serve as prognostic markers for overall survival in glioma patients.

### Establishment and Validation of the Seven-Gene Prognostic Signature

**FIGURE 6:**
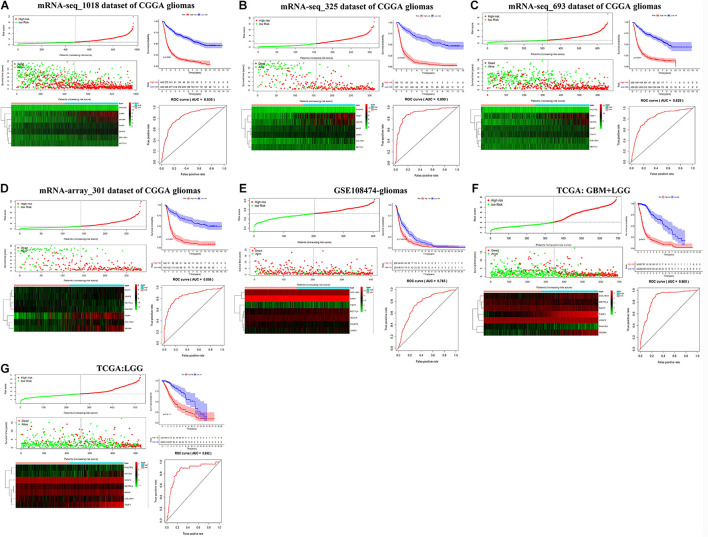
Kaplan–Meier plot, risk score, and time-dependent ROC analyses for the seven-gene signature in glioma. **(A–G)** Risk score, heatmap of mRNA expression, Kaplan–Meier curve, and time-dependent ROC analysis of the seven-gene signature in seven sets of CGGA mRNA datasets, including mRNA-seq_1018, mRNA-seq_325, mRNA-seq_693, and mRNA-array_301, GSE108474, TCGA-GBM-LGG, and TCGA-LGG.

We then established a prognostic gene signature for overall survival in glioma patients by Lasso-penalized Cox analysis (Supplementary Figure S7). Firstly, METTL3 and its corresponding validated targets (NASP, TIMP1, U2AF2, COL18A1, PHLPP2, and VEGFA) were determined and subsequently utilized to construct a prognostic gene signature in the mRNA-seq_1018 dataset of CGGA. We then calculated the seven-gene-based risk score [Risk score = 0.1065 ^∗^ Exp**NASP** + 0.1099 ^∗^ Exp**TIMP1**+ 0.5640 ^∗^ Exp**U2AF2** + 0.1682 ^∗^ Exp**VEGFA** + (−0.2620) ^∗^ Exp**COL18A1** + (−0.1799) ^∗^ Exp**METTL3** + (−0.4656) ^∗^ Exp**PHLPP2**] for each patient and identified the optimal cutoff for the risk score. Next, the prognostic ability of the seven-gene signature was assessed by the time-dependent ROC and Kaplan–Meier curve. We found that high-risk glioma patients have a significantly shorter overall survival than low-risk patients (*p* < 0.0001) (Figure 6A). Similar procedures were conducted in CGGA, TCGA, and GSE108474 datasets, and the above results were consistently validated in three CGGA datasets and GSE108474 of glioma, the merged TCGA-GBM and LGG dataset, and the TCGA-LGG dataset (Figures 6B–G). Moreover, the good AUC (Area under the ROC curve) for overall survival was also consistently validated in the seven sets of mRNA datasets, which was 0.838, 0.899, 0.828, 0.856, 0.765, 0.865, and 0.852, respectively (Figures 6A–G). Collectively, our results indicated that the seven-gene signature has a good capability for predicting survival in glioma.

**FIGURE 7:**
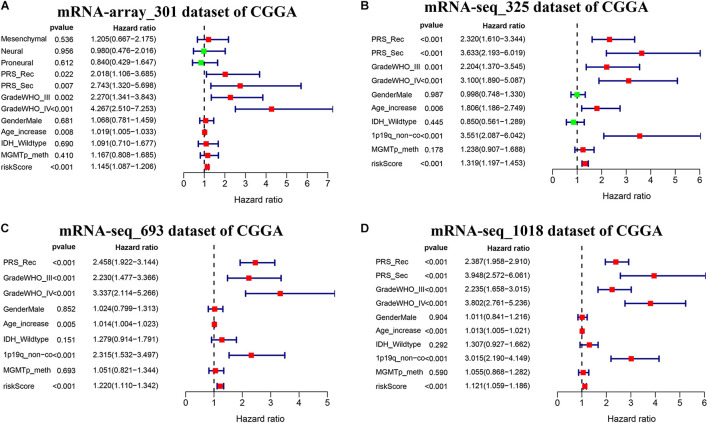
Effects of the risk score and clinical features on the prognosis of glioma patients. **(A–D)** Cox multivariate analyses of clinical features and risk score, and overall survival in four sets of CGGA mRNA datasets, including mRNA-array_301, mRNA-seq_325, mRNA-seq_693, and mRNA-seq_1018.

We further conducted multivariate Cox proportional hazard regression analyses for four CGGA datasets with complete information including age, gender, PRS type, WHO grade, IDH_mutation status, 1p19q_codeletion status, and MGMTp_methylation status to define which clinical factors could be used as prognostic indicators for the overall survival of glioma. As shown in Figures 7A–D and Supplementary Table S9, the analyses for all datasets revealed that risk score calculated from the seven-gene signature is significantly correlated with the poor overall survival of glioma (HR > 1, *p* < 0.001). We observed that the recurrent or secondary patients in four datasets consistently showed a poor overall survival than the primary patients (HR > 1, *p* < 0.05). Similarly, the age increase of patients in four datasets also consistently exhibited a poor overall survival (HR > 1, *p* < 0.01). Compared to patients with WHO II grade in all datasets, patients with WHO III or IV grade had a shorter overall survival (HR > 1, *p* < 0.01). In three datasets with 1p19q_codeletion information, 1p19q_non-codeletion showed a consistent relationship with poor overall survival of glioma, compared with 1p19q_codeletion (HR > 2, *p* < 0.01) (Figures 7B–D). These results demonstrated that risk score calculated from the seven-gene signature, age increase, recurrent and secondary status, the high WHO grade, and 1p19q_non-codeletion were independent prognostic factors for overall survival.

### Identification of Key m6A RNA Methylation Regulators and Targets for 81 Anticancer Drug Susceptibility in Cancer Cell Lines

**FIGURE 8:**
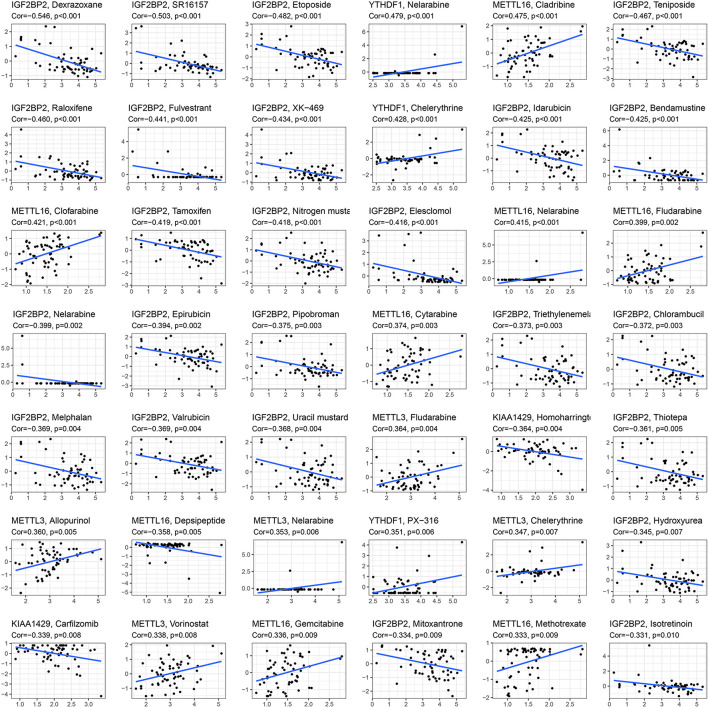
Identification of five m6A RNA methylation regulators for anticancer drug responses in cancer cell lines. The *x*-axis represents gene expression level and the *y*-axis represents drug sensitivity score.

To explore whether key glioma-related m6A RNA methylation regulators (KIAA1429, METTL16, METTL3, IGF2BP2, and YTHDF1) and targets (COL18A1, NASP, PHLPP2, TIMP1, U2AF2, and VEGFA) were associated with anticancer drug responses, we performed a correlation analysis between the expressions of these genes and drug susceptibility using CellMiner database and R packages impute, limma, ggplot2, and ggpubr. We determined 81 anticancer drug responses influenced by these genes. For m6A RNA methylation regulators, we found that YTHDF1, METTL3, and METTL16 expressions are positively linked to 15 drug susceptibility, while only METTL16 expression is negatively associated with Depsipeptide susceptibility. In addition, KIAA1429 and IGF2BP2 expressions showed a widely negative correlation with 26 drug responses (Figure 8). For six targets of METTL3, the expression of NASP had a broadly positive relevance with 21 anticancer drug responses, and COL18A1, PHLPP2, TIMP1, U2AF2, and VEGFA expressions showed positive or negative relationships with 45 anticancer drug responses (Supplementary Table S10).

## Discussion

Glioma remains a main challenge for public health worldwide with poor prognosis (Du et al., 2020). Increasing strong evidence suggested that multiple m6A RNA methylation regulators can regulate the self-renewal, growth and progression, and invasion of glioma cell by regulating mRNA expression levels of their target genes, which provides a reliable support for the m6A regulator–target gene axes as some specific and novel therapeutic targets and clinical prognostic signature in glioma. In the current study, we performed systematic analyses for m6A RNA methylation regulators and their validated and potential targets in multiple glioma datasets. Our results suggested that IGF2BP2, KIAA1429, METTL16, and METTL3, as well as 208 targets are involved in the occurrence of glioma, GBM, and LGG. YTHDF1 and 78 targets involved the occurrence of glioma and GBM, not LGG.

The association analysis and miRNA–mRNA regulatory network analysis indicated that the cross-talk between m6A RNA methylation regulators and their targets may play critical roles in the occurrence of glioma. Function enrichment analyses indicated that KIAA1429, METTL16, METTL3, IGF2BP2, and YTHDF1 mainly regulate the methylation, destabilization, and metabolic process of RNA and mRNA, and influence the growth and development, differentiation, transport, and ensheathment of multiple brain cell types. Our analysis suggested that Notch signaling pathway (including key genes METTL3, DLL3, HES1, and NOTCH3) closely implicated with tumorigenesis and cancer development (Li et al., 2017) was found to be involved in glioma. Subsequently, we explored the potential regulation interactions between them utilizing correlation analysis in glioma, GBM, and LGG. We found that METTL3 is positively associated with DLL3, HES1, and NOTCH3 expressions in glioma, especially this association that was identified in both mRNA and protein levels of GBM, which provides a support for previous observations that silencing METTL3 can reduce the transcript and protein levels of DLL3, HES1, and NOTCH3 in the glioblastoma stem cell line and increase cell apoptosis (Visvanathan et al., 2019). To further validate the influence of METTL3 in Notch signaling pathway and glioma occurrence, we knocked down METTL3 in two GBM cell lines. qRT-PCR and Western blot analyses showed that METTL3 knockdown significantly decreases the mRNA and protein levels of NOTCH3, DLL3, and HES1. CCK8 assay showed that METTL3 knockdown inhibits glioblastoma cell proliferation at 72 h after transfection. Collectively, these findings and other previous report demonstrated that METTL3 can activate Notch pathway and facilitate glioma occurrence through regulating its direct targets NOTCH3, DLL3, and HES1, and Notch pathway genes may serve as the potential treatment targets for glioma.

To determine the prognostic value of five m6A RNA methylation regulators and their 286 targets in glioma, we performed survival and univariate Cox regression analyses in multiple mRNA datasets. The results showed that 181 genes are validated to be consistently correlated with overall survival of glioma patients. Glioma patients with high expressions of METTL3, IGF2BP2, VEGFA, TIMP1, HES1, COL18A1, TK1, CENPK, DNAJB11, NASP, and U2AF2 had a shorter overall survival, while glioma patients with high expressions of PHLPP2, ATP6V1A, and DLL3 showed a good overall survival, indicating that these genes could serve as prognostic markers for overall survival in glioma patients. Then, Lasso-penalized Cox analysis, time-dependent ROC, Kaplan–Meier curve, and multivariate Cox proportional hazard regression analysis in seven sets of mRNA datasets indicated that the seven-gene signature comprising METTL3, COL18A1, NASP, PHLPP2, TIMP1, U2AF2, and VEGFA has a good capability for predicting survival in glioma. In addition, our results demonstrated that the age increase, recurrent and secondary status, the high WHO grade, and 1p19q_non-codeletion were independent risk factors for poor overall survival of glioma patients.

As summarized in Supplementary Table S3, five m6A RNA methylation regulators KIAA1429, METTL16, METTL3, IGF2BP2, and YTHDF1 were shown to be implicated in the growth and proliferation, colony formation ability, migration, invasion, and apoptosis of tumor cells by altering the m6A methylation, transcript, and protein levels of their targets. For m6A writers, METTL3 was reported to be upregulated in 13 types of cancers and serve as an oncogene in which METTL3 can regulate the differentiation and apoptosis, survival, and metastasis of cancers containing glioma *via* influencing the translation, stability, and AKT pathway of oncogenes, tumor suppressors, and miRNAs. In endometrial cancer, it was reported to be downregulated and to modulate the expression of members of the AKT pathway and inhibit cell proliferation (Cai et al., 2018; Visvanathan et al., 2018; Miao et al., 2019; Peng et al., 2019; Li et al., 2020; Liang et al., 2020; Nombela et al., 2021). KIAA1429 was reviewed to be upregulated in 12 types of cancers and to be downregulated in 4 cancer types (Zhu et al., 2021). METTL16 was found to be overexpressed in colorectal cancer and to be underexpressed in hepatocellular carcinoma, affecting activation of multiple metabolic pathways (Liu et al., 2019; Wang et al., 2020). For m6A readers, IGF2BP2 expression was reported to be enhanced, and promote proliferation, invasion, and migration of tumor in eight tumors including glioma (Kessler et al., 2017; Huang et al., 2019; Wang and Chen, 2021). YTHDF1 was reported to be overexpressed in ovarian cancer, colorectal cancer, and breast cancer and facilitate tumorigenesis and metastasis (Bai et al., 2019; Liu et al., 2020; Nombela et al., 2021).

In our study, six validated targets of METTL3 were observed to be associated with the occurrence and survival of multiple cancers including glioma, which is similar to previous reports that NASP, TIMP1, U2AF2, and VEGFA were upregulated in other cancers, and TIMP1 had a broad correlation with the progression or poor prognosis of multiple cancers, while PHLPP2 was downregulated in colorectal cancer and colon cancer (Ali-Fehmi et al., 2010; Guo et al., 2015; Jackson et al., 2017; Cao et al., 2019; Li J. et al., 2019; Wu et al., 2019, 2020; Kong et al., 2020; Supplementary Table S11). Collectively, m6A RNA methylation regulators KIAA1429, METTL16, METTL3, IGF2BP2, and YTHDF, and targets NASP, TIMP1, U2AF2, COL18A1, and VEGFA could serve as oncogenes in glioma, while PHLPP2 is a tumor suppressor. Furthermore, these genes were identified to influence 81 anticancer drug responses, which further contribute to the early phase clinical trials of anticancer drug development.

## Conclusion

In conclusion, m6A RNA methylation regulators KIAA1429, METTL16, METTL3, IGF2BP2, and YTHDF1, as well as their targets may play a critical role in the occurrence of glioma, which may be beneficial to therapeutic customization and clinical decision-making. METTL3 can activate Notch pathway and facilitate glioma occurrence through regulating its direct targets NOTCH3, DLL3, and HES1, and Notch pathway genes may serve as the potential treatment targets for glioma. Our study firstly established a seven-gene signature comprising METTL3, COL18A1, NASP, PHLPP2, TIMP1, U2AF2, and VEGFA with a good capability for predicting survival in glioma.

## Data Availability Statement

Publicly available datasets were analyzed in this study. This data can be found here: The datasets that support the findings of our study are openly available in The Cancer Genome Atlas (TCGA) of UCSC Xena at https://xena.ucsc.edu/, Chinese Glioma Genome Atlas (CGGA) at http://www.cgga.org.cn/, Clinical Proteome Tumor Analysis Consortium (CPTAC) at https://proteomics.cancer.gov/programs/cptac, GEPIA 2 database (http://gepia2.cancer-pku.cn/), and GSCALite (http://bioinfo.life.hust.edu.cn/web/GSCALite/). nci60_RNA-seq and compound activity data DTP NCI-60 was downloaded from CellMiner database 2.5 at https://discover.nci.nih.gov/cellminer/loadDownload.do.

## Author Contributions

XH and LX designed the research. PC, TW, and XH drafted the manuscript and revised the manuscript. XH, HL, XG, and LT performed the analyses. XH, WL, AC, HW, MH, DD, and ZL participated in data consolidation and plotting. All authors read and approved the final manuscript.

## Conflict of Interest

The authors declare that the research was conducted in the absence of any commercial or financial relationships that could be construed as a potential conflict of interest.

## Publisher’s Note

All claims expressed in this article are solely those of the authors and do not necessarily represent those of their affiliated organizations, or those of the publisher, the editors and the reviewers. Any product that may be evaluated in this article, or claim that may be made by its manufacturer, is not guaranteed or endorsed by the publisher.

## Footnotes

1
https://xena.ucsc.edu/

2
http://www.cgga.org.cn

3
https://www.ncbi.nlm.nih.gov/gds/

4
https://proteomics.cancer.gov/programs/cptac

5
http://m6a2target.canceromics.org

6
http://bioinfo.life.hust.edu.cn/GSCA/#

7
http://www.string-db.org/

8
http://starbase.sysu.edu.cn/

## Supplementary Material

Supplementary Figure S1 The workflow and scheme of this study.

Supplementary Figure S2 Validation of expression profiles of the 24 validated targets in GBM and LGG by the GEPIA2 database.

Supplementary Figure S3 Biological functions of all differential expressed m6A RNA methylation regulators and their validated and potential targets. **(A)** Identification of biological process (BP), molecular function (BP), and cell composition (CC). **(B)** Identification of signaling pathways.

Supplementary Figure S4 The correlation analyses between genes, microRNAs, and cancer-related pathways by Gene Set Cancer Analysis (GSCA) (http://bioinfo.life.hust.edu.cn/GSCA/#/expression) and starBase v3.0 databases (http://starbase.sysu.edu.cn/). **(A)** Identification of genes correlated with activation or inhibition of multiple pathways. **(B)** The high expressions of genes activate or inhibit eight cancer-related pathways in GBM, containing TSC/mTOR, hormone AR, hormone ER, DNA damage response, RTK, EMT, apoptosis, and cell cycle. **(C)** The potential miRNA-mRNA regulatory network of five m6A RNA methylation regulators.

Supplementary Figure S5 Knockdown efficiency of siRNAs in METTL3. **(A,B)** The knockdown efficiency of METTL3 gene in U118 cells by three small interfering RNAs (siRNAs) were determined by qRT-PCR and Western blot (*n* = 3). **(C,D)** The knockdown efficiency of METTL3 gene in U78 cells by three small interfering RNAs (siRNAs) were determined by qRT-PCR and Western blot (*n* = 3). Data was represented as mean ± SEM. ^∗^Represented comparison with siCtrl, ^∗^*P* < 0.05; ^∗∗^*P* < 0.01; ^∗∗∗^*P* < 0.001.

Supplementary Figure S6 Validation of m6A RNA methylation regulators or validated targets associated with overall survival of glioma patients in 2 other sets of CGGA mRNA datasets including mRNA-seq_325 **(A)** and mRNA-seq_693 **(B)**.

Supplementary Figure S7 Lasso-penalized Cox analysis of 7 m6A RNA methylation regulators or validated targets associated with overall survival in glioma.

Supplementary Table S1 METTL3 siRNA sequences and METTL3, DLL3, HES1, NOTCH3, and GADPH primer sequences used for qRT-PCR.

Supplementary Table S2 The clinical characteristics for TCGA, CGGA, GSE16011, GSE108474, and CPTAC datasets.

Supplementary Table S3 Differentially expressed m6A RNA methylation regulators and their validated and potential targets in glioma.

Supplementary Table S4 The validated and potential targets of m6A RNA methylation regulators were obtained from m6A2Target database (http://m6a2target.canceromics.org).

Supplementary Table S5 Differentially expressed m6A RNA methylation regulators and their validated and potential targets in pan-cancers by GSCA.

Supplementary Table S6 Biological functions of all differential expressed m6A RNA methylation regulators and their targets.

Supplementary Table S7 The identified correlation between 5 m6A RNA methylation regulators and targets in glioma, GBM, and LGG.

Supplementary Table S8 Identification and validation of 181 m6A RNA methylation regulators or targets associated with overall survival of cancer patients, including glioma, LGG, and GBM in 4 sets of CGGA mRNA datasets and GSCA database.

Supplementary Table S9 Multivariate Cox analyses of clinical features, risk score, and overall survival in 4 sets of CGGA mRNA datasets.

Supplementary Table S10 Identification of 11 m6A RNA methylation regulators or targets for 81 anticancer drug responses in cancer cell lines.

Supplementary Table S11 The summary of role of seven-gene signature in other cancers.

## Abbreviations

LGG: low-grade gliomas
GBM: glioblastoma
TCGA: The Cancer Genome Atlas
CPTAC: Clinical Proteome Tumor Analysis Consortium
CGGA: Chinese Glioma Genome Atlas
GEPIA2: Gene expression profiling interactive analysis 2
METTL3: Methyltransferase-Like 3
METTL16: Methyltransferase-Like 16
KIAA1429 (VIRMA): Vir-Like M6A Methyltransferase Associated
IGF2BP2: Insulin-Like Growth Factor 2 MRNA Binding Protein 2
YTHDF1: YTH N6-Methyladenosine RNA Binding Protein 1
COL18A1: Collagen Type XVIII Alpha 1 Chain
NASP: Nuclear Autoantigenic Sperm Protein
PHLPP2: PH Domain and Leucine Rich Repeat Protein Phosphatase 2
TIMP1: TIMP Metallopeptidase Inhibitor 1
U2AF2: U2 Small Nuclear RNA Auxiliary Factor 2
VEGFA: Vascular Endothelial Growth Factor A
DLL3: Delta-Like Canonical Notch Ligand 3
HES1: Hes Family BHLH Transcription Factor 1
NOTCH3: Notch Receptor 3.
