# Supplementary figures and images for: Identification of the Role and Clinical Prognostic Value of Target Genes of m6A RNA Methylation Regulators in Glioma
Source: Front Cell Dev Biol. 2021 Sep 13;9:709022. doi: 10.3389/fcell.2021.709022 (PMC8473691; doi:10.3389/fcell.2021.709022)

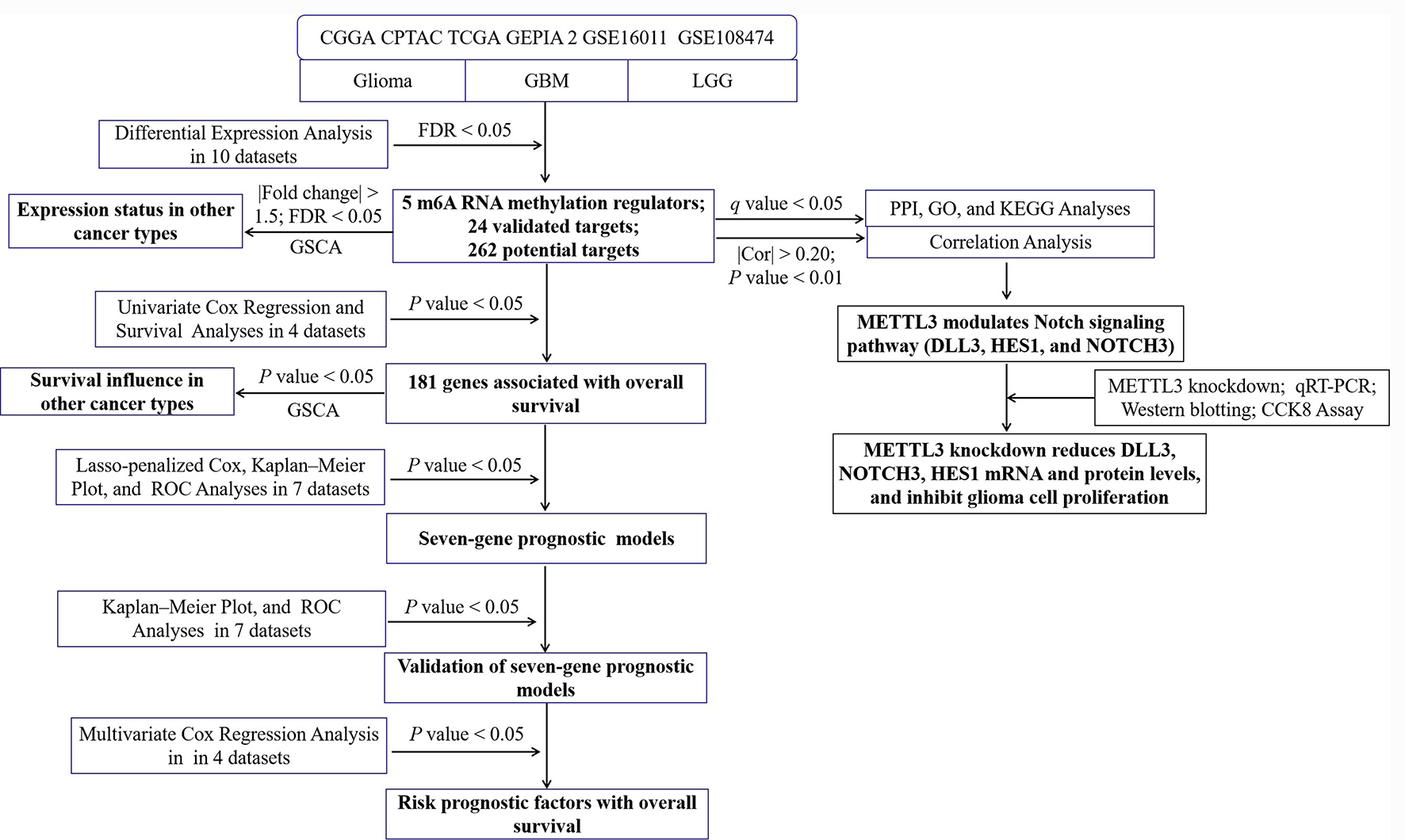

Supplement: Supplementary Figure S1 — The workflow and scheme of this study. [file Image_1.JPEG]

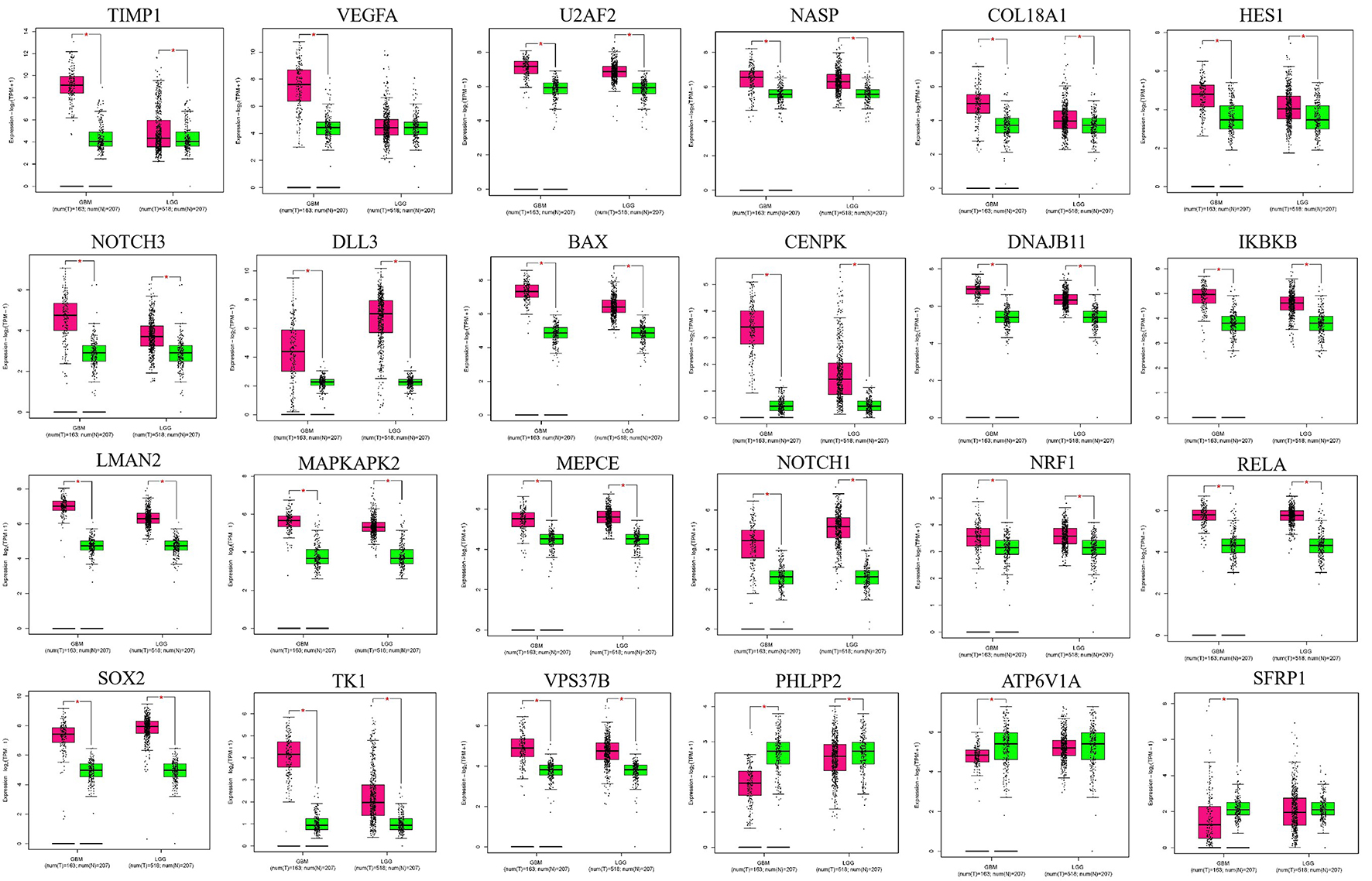

Supplement: Supplementary Figure S2 — Validation of expression profiles of the 24 validated targets in GBM and LGG by the GEPIA2 database. [file Image_2.JPEG]

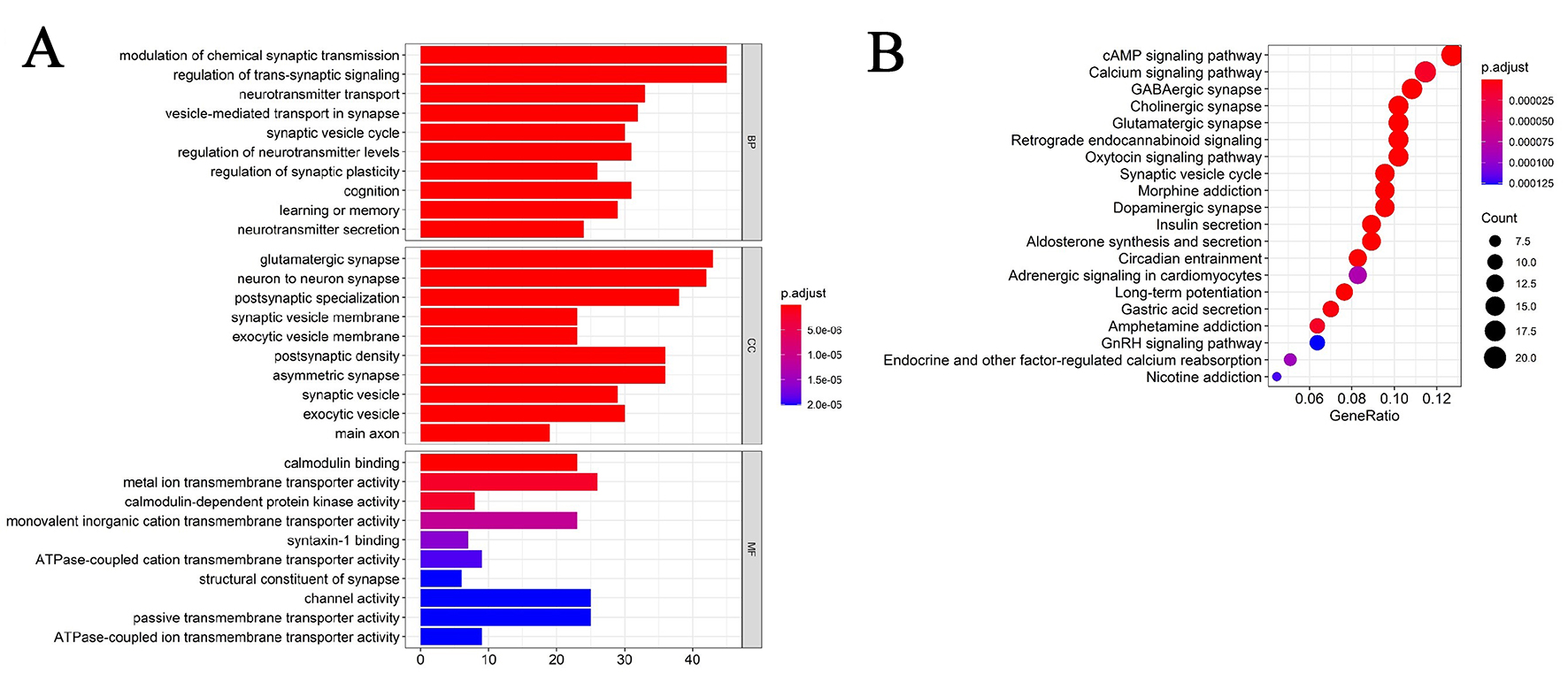

Supplement: Supplementary Figure S3 — Biological functions of all differential expressed m6A RNA methylation regulators and their validated and potential targets. (A) Identification of biological process (BP), molecular function (BP), and cell composition (CC). (B) Identification of signaling pathways. [file Image_3.JPEG]

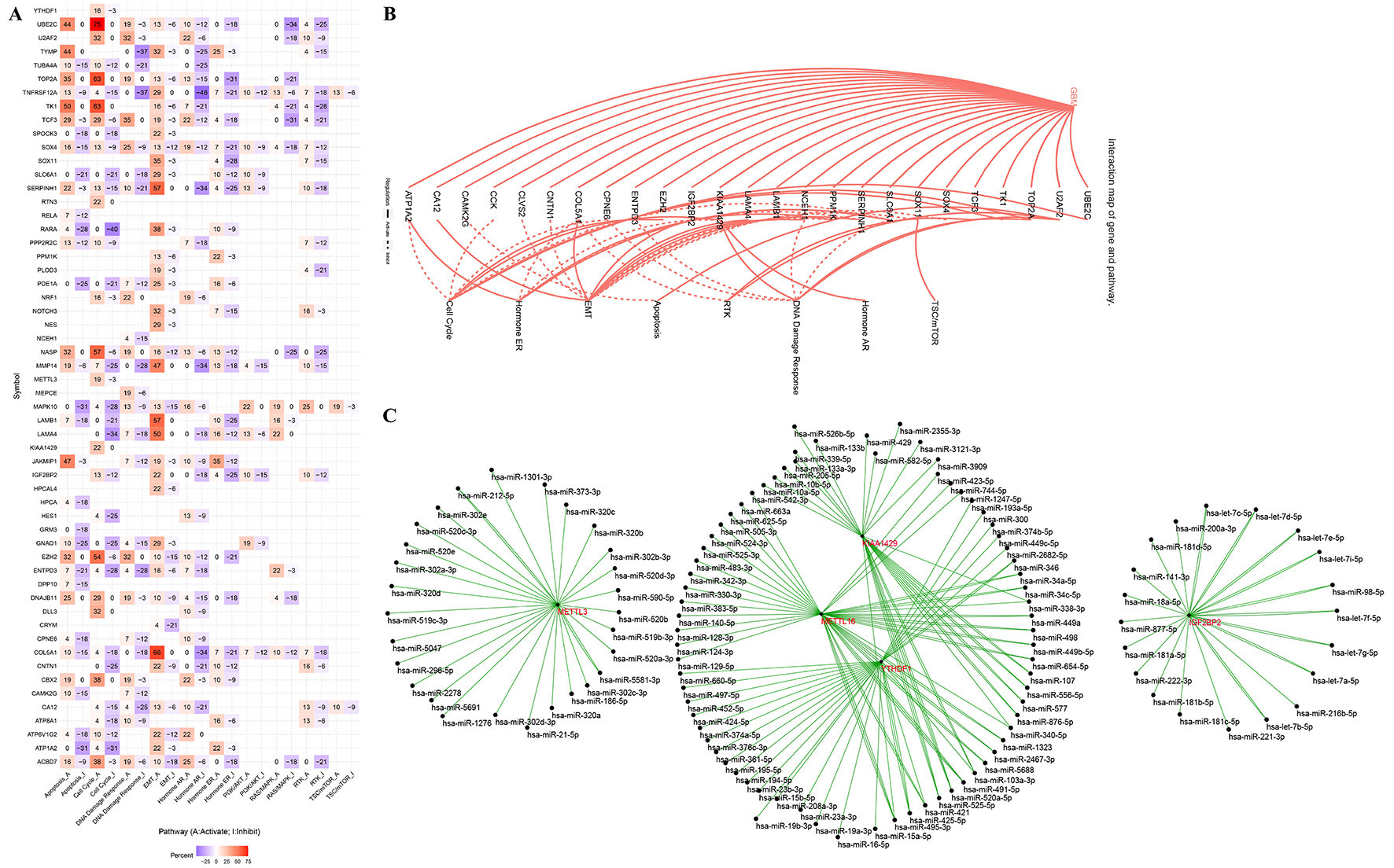

Supplement: Supplementary Figure S4 — The correlation analyses between genes, microRNAs, and cancer-related pathways by Gene Set Cancer Analysis (GSCA) (http://bioinfo.life.hust.edu.cn/GSCA/#/expression) and starBase v3.0 databases (http://starbase.sysu.edu.cn/). (A) Identification of genes correlated with activation or inhibition of multiple pathways. (B) The high expressions of genes activate or inhibit eight cancer-related pathways in GBM, containing TSC/mTOR, hormone AR, hormone ER, DNA damage response, RTK, EMT, apoptosis, and cell cycle. (C) The potential miRNA-mRNA regulatory network of five m6A RNA methylation regulators. [file Image_4.JPEG]

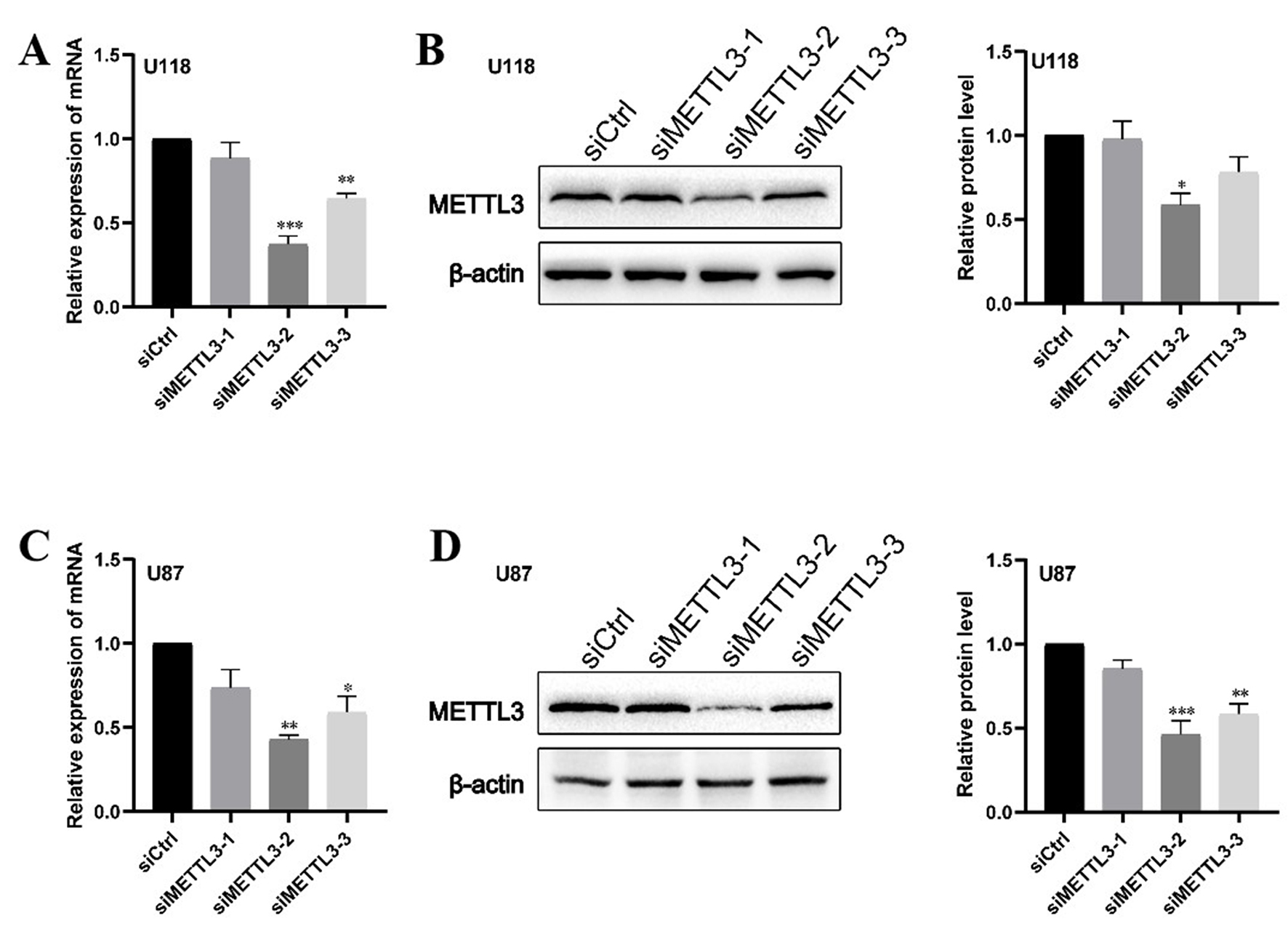

Supplement: Supplementary Figure S5 — Knockdown efficiency of siRNAs in METTL3. (A,B) The knockdown efficiency of METTL3 gene in U118 cells by three small interfering RNAs (siRNAs) were determined by qRT-PCR and Western blot (n = 3). (C,D) The knockdown efficiency of METTL3 gene in U78 cells by three small interfering RNAs (siRNAs) were determined by qRT-PCR and Western blot (n = 3). Data was represented as mean ± SEM. ∗Represented comparison with siCtrl, ∗P < 0.05; ∗∗P < 0.01; ∗∗∗P < 0.001. [file Image_5.JPEG]

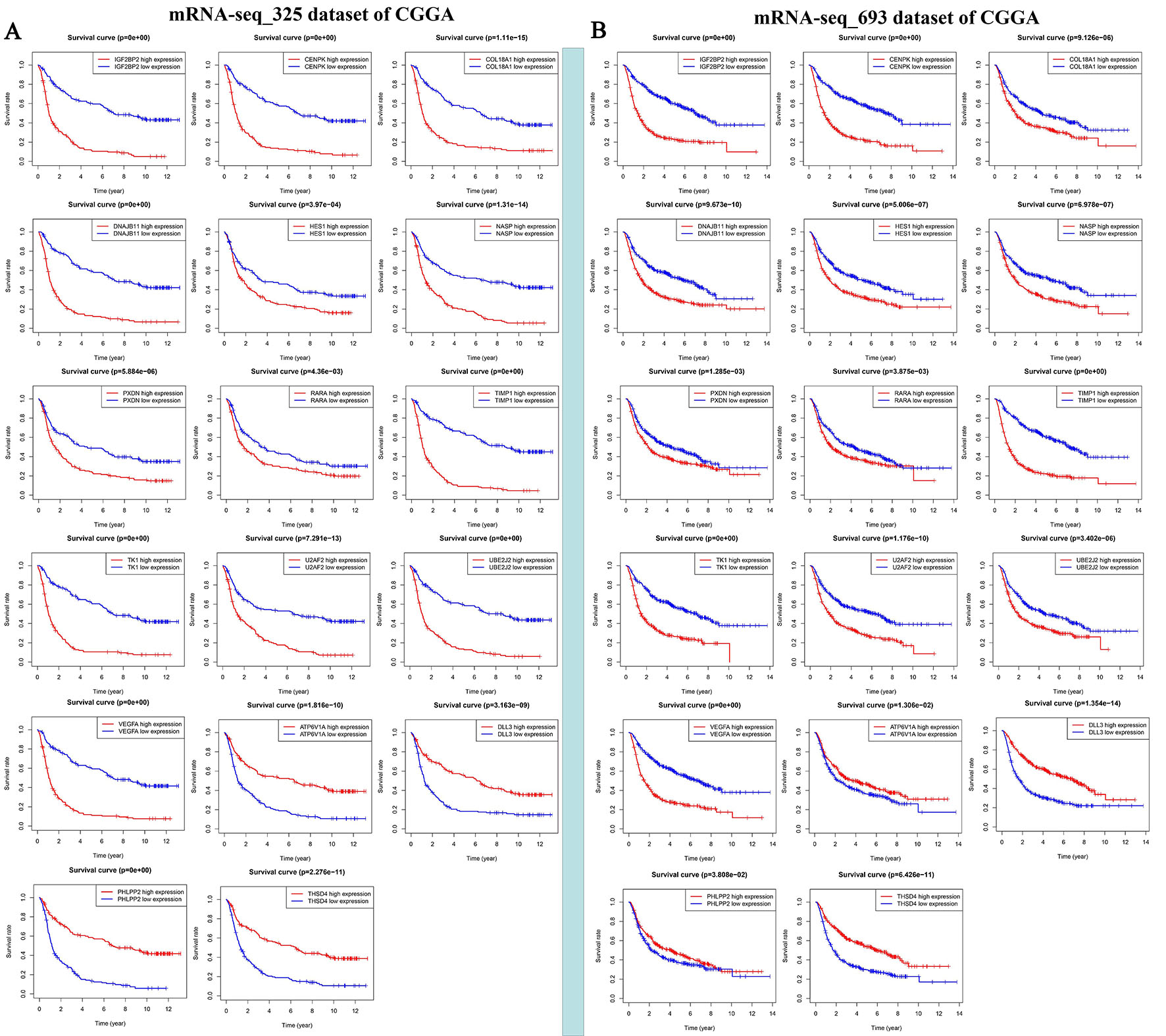

Supplement: Supplementary Figure S6 — Validation of m6A RNA methylation regulators or validated targets associated with overall survival of glioma patients in 2 other sets of CGGA mRNA datasets including mRNA-seq_325 (A) and mRNA-seq_693 (B). [file Image_6.JPEG]

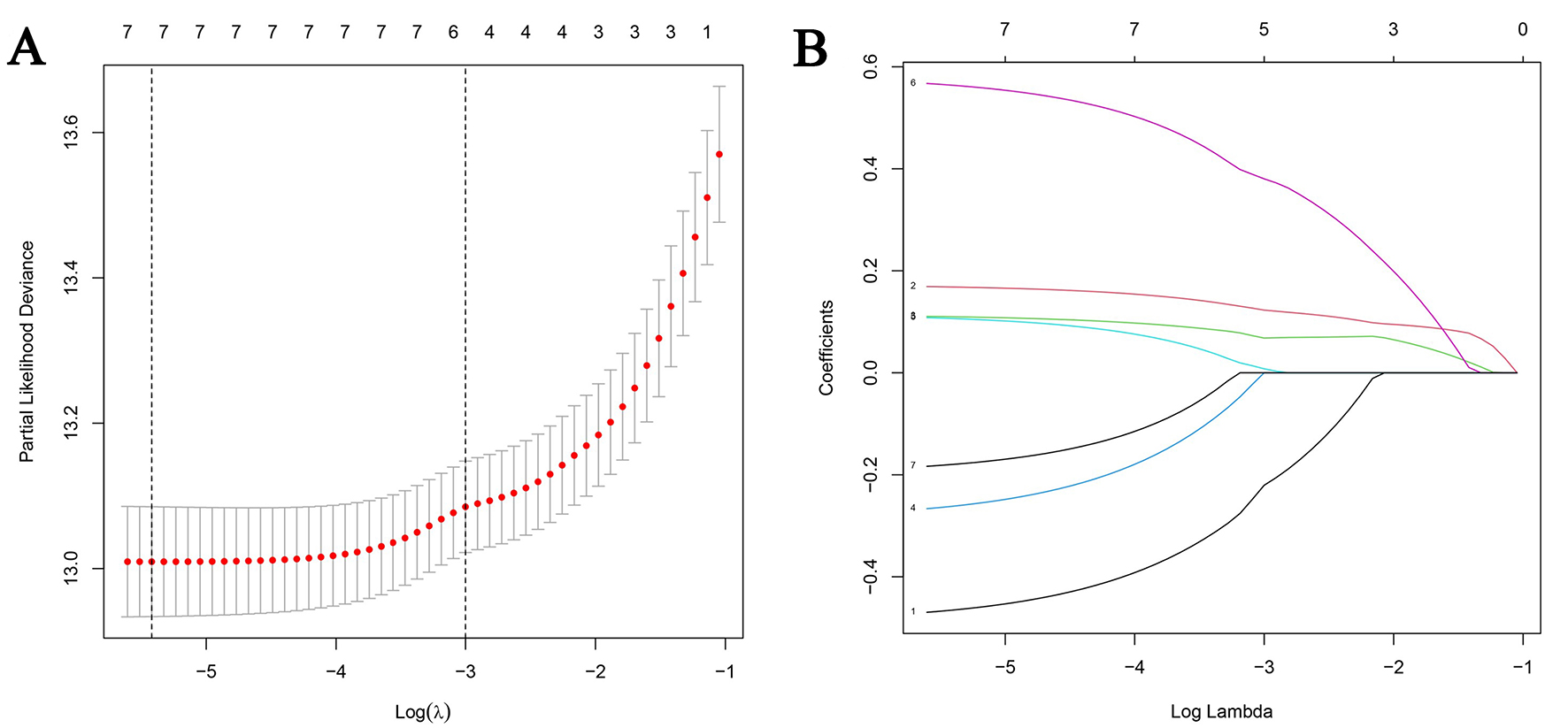

Supplement: Supplementary Figure S7 — Lasso-penalized Cox analysis of 7 m6A RNA methylation regulators or validated targets associated with overall survival in glioma. [file Image_7.JPEG]
